# Quick Transition to One Day Length of Stay after Hip and Knee Arthroplasty Using a Digital Follow-Up Tool during COVID-19: A Retrospective Comparative Study

**DOI:** 10.3390/healthcare11182516

**Published:** 2023-09-11

**Authors:** Philippe Van Overschelde, Wouter Van Lysebettens, Julien Lebleu, Andries Pauwels, Sebastien Parratte

**Affiliations:** 1 Hip and Knee Unit, 9830 Ghent, Belgium; vanoverschelde@gmail.com (P.V.O.);; 2 moveUP, Cantersteen 47, 1000 Brussels, Belgium; andries@moveup.care; 3 International Knee and Joint Centre, Abu Dhabi 46705, United Arab Emirates; sebastien.parratte@gmail.com; 4 Locomotion Institute, Aix-Marseille University, 13009 Marseille, France

**Keywords:** telerehabilitation, COVID-19, arthroplasty, outpatient surgery

## Abstract

The COVID-19 pandemic highlighted the need for efficient use of hospital infrastructure. The hypothesis was that a rapid shift to outpatient surgery after hip or knee arthroplasty could be implemented without compromising quality of care. The aim of this study was to assess the safety, pain management and patient-reported outcomes before and after the implementation of an accelerated discharge program using a digital follow-up tool. A retrospective cohort design was used to compare 97 patients who received primary total hip or knee arthroplasty during the pandemic (early discharge) to comparable 194 pre-pandemic patients (normal discharge). Both cohorts had the same inclusion criteria and were closely monitored using the digital follow-up tool. The accelerated discharge program reduced length of stay from a median of 3 days (before the pandemic) to a median of 1 day (during the pandemic) (*p* < 0.001). The complication rate of 2% was the same for both groups (*p* > 0.05). Patient-reported outcomes for matched samples of hip (n = 100) and knee (n = 82) arthroplasty patients were similar before, at 6 weeks and 3 months after surgery for both groups (*p* > 0.05). There were no differences in pain and medication consumption for the first 6 weeks (*p* > 0.05). This study demonstrates that reducing length of stay from three to one night after total knee or hip arthroplasty, with the help of a digital follow-up tool, results in a stable rate of complications, readmission, and comparable clinical outcomes, while reducing the socio-economic burden on the health system.

## 1. Introduction

The appropriate and efficient use of hospital infrastructure is important for health care providers and payors [1]. Reduction of length of stay (LOS) without compromising the quality of care is a trend observed in all specialties [2]. The COVID-19 pandemic added pressure on hospital infrastructure and limited the access to patients requiring an elective surgery such as hip or knee arthroplasty. The number of arthroplasties during the pandemic decreased significantly in the United States [3], Belgium [4] and Poland [5].

In Europe, hospitals were asked to preserve bed capacity to be able to accept patients with COVID-19. To be able to keep operating on patients, surgeons had to very quickly shift to a very short length of stay for their patients [5]. In Poland, for example, the mean time of hospital stay following hip or knee arthroplasties was 23% shorter [5].

It has been shown that a shorter length of stay provides multiple benefits for the patient, such as lower infection rates, faster return to activities of daily living and reduction in thrombo-embolic events [6,7,8,9,10]. The concept of fast-track surgery and ERAS (Enhanced Recovery After Surgery) [11,12] have been well described in the literature [13]. In the US, outpatient surgeries became standard of care as more and more arthroplasties were performed in outpatient surgery centers. To achieve shorter length of stay, the whole pathway needs to be optimized, including patient education, effective multimodal pain management, accelerated rehabilitation and the monitoring of complications and outcomes. To set up such changes in a surgeon’s practice usually requires time, preparation and teamwork, with progressive implementation of this new pathway. During the pandemic, clinical teams have had to switch very quickly to accelerate discharge. In this context, digital solutions consisting of a remote patient support tool can be useful to support teams [14,15]. Digital solutions has the potential to increase access to care [16], provide real-time data and feedback on patient evolution, and deliver equivalent outcomes compared to standard of care [17,18]. Digital solutions typically contain education modules to better prepare patients for the intervention and to answer the most frequent questions post-surgery. Additionally, they provide a data collection tool to monitor patients remotely and intervene in case of need. Finally, some propose a rehabilitation program to guide patients in their recovery. Many hospital systems are implementing digital solutions in their standard of care, in order to gain efficiencies and better monitor their processes. The digital solution used in this study has been shown to be an effective digital solution in a separate study, offering quality of care in a cost-efficient way [14]. 

It was our hypothesis that an accelerated discharge program, implemented via this digital follow-up tool, could reduce length of stay from three to one night without compromising the clinical results. Therefore, the goals of this study were to compare (1) safety as measured by the rate of complications and readmissions, (2) pain management and control as measured by remote monitoring of the visual analogic pain scale and the medication consumption and (3) the patient-reported outcomes as measured using the digital patient evaluation platform. 

## 2. Materials and Methods

In this retrospective comparative cohort study, 292 patients operated on for an elective primary total hip or total knee arthroplasty performed by a single surgeon in the same institution (PVO) between October 2018 and February 2021 were included. All the patients were included in a consecutive manner when meeting the inclusion criteria and respecting the exclusion criteria. The ethics committee of Universitair Ziekenhuis Antwerp (UZA) approved the study protocol and each patient provided written informed consent to the use of their anonymized data for scientific use.

The inclusion criteria were primary hip or knee arthroplasty, and use of the digital app (moveUP solution, Brussels, Belgium). The exclusion criteria were revision hip or knee, and incomplete use of the digital app. The study group (pandemic) had elective knee or hip arthroplasties between May 2020 and February 2021. Patients of the control group (pre-pandemic) were operated on between October 2018 and April 2020. The classically described indication–contraindications for THA and TKA outpatient surgeries were used for the selection of the patients during the pandemic [19].

In order to minimize potential biases and enhance the comparability between the study and control groups, propensity score matching was employed as part of the analysis. Propensity scores were calculated for each patient based on their demographic and clinical characteristics, including age (±5), gender, BMI (Body Mass Index) (±3), type of surgery, pre-operative PROMS (Patient-reported Outcome Measures), and comorbidities measured with the ASA classification (American Society of Anesthesiologists physical status classification system) [20,21]. Only ASA 1 and 2 were included. Subsequently, patients from the study group were matched to corresponding patients from the control group based on these propensity scores. This process ensured that individuals with similar profiles were paired together, thereby reducing the impact of confounding variables. The effectiveness of the propensity score matching was confirmed by evaluating the standardized mean differences before and after matching, demonstrating a significant improvement in the balance between the groups (Appendix A). 

moveUP Therapy (moveUP, Belgium) is registered as a medical device and uses a virtual platform for digital follow-up based on objective and subjective patient data, combined with personalized interaction between a therapist and the patient. The treatment is continuously adapted and personalized automatically and clinically according to the patient’s needs.

Before surgery, patients were prepared through an individualized education session at the hospital to explain the pathway and how to use the app. They were also instructed to follow the notifications of the app and to learn from the tutorial offered in the app. The pandemic group was informed, via the digital solution, on the fast discharge protocol and the follow-up they would receive via the tool after discharge. The anesthetic protocol and the pain management protocol were standardized based on the latest recommendations of the literature, and were similar in both groups, though the pandemic group had quicker transfers to their room and mobilization. Similar modern multimodal pain management protocols were used in both groups. The same standardized DVT prophylaxis protocol was used in both groups based on international recommendations. 

The discharge criteria for both groups were a combination of VAS pain < 5 (visual analogic scale) with medication, flexion >90 degrees, able to walk with walking aids and to do transfers, dry wounds, and readiness/confidence of the patient to go home. The pandemic was a strong motivator for patients to return home quicker, supported by the follow-up they would receive via the digital app. 

**Figure 1:**
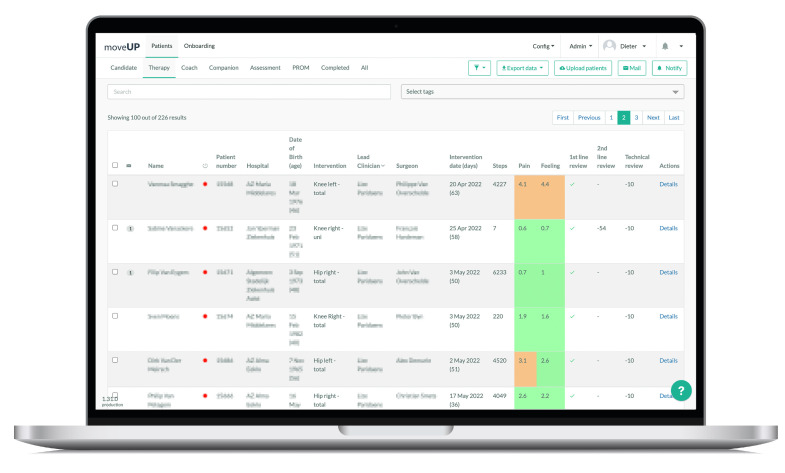

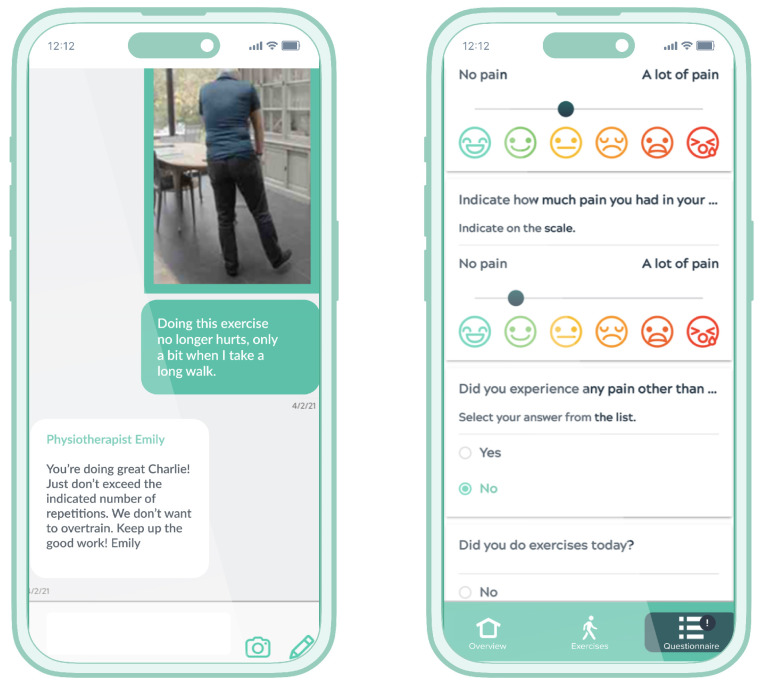
Medical dashboard and patient app screenshots of the daily survey and the secured messaging system.

After discharge, the follow-up of patients was performed using the application. The clinical team was trained on the fast discharge program and the attention points of early discharge monitoring via digital follow-up tool. The clinical team has insight on a broad range of data to control their patient’s pre and post-surgery progress: data on physical activity, pain levels, medication use, exercise adherence, patient-reported outcome measures, pictures of the surgical wound and videos of range of movement are collected, amongst others. Patients received regular information about their recovery status, personalized and adapted physiotherapy protocol (PT), personalized pain management protocols (Figure 1). The patient was able to communicate with the moveUP team through a secured chat messaging system. Both groups received the same support, adapted PT exercises and pain management program via the application. The frequency of use of the application by the patients and the care team was the same in both groups. The only difference is that the care team started managing the treatment of the pandemic group earlier as the discharge occurred earlier. 

### 2.1. Data Collection

The length of stay (LOS) was assessed through a question in the app—“how many nights did you stay in hospital?”—and a cross-checking process was performed with the data from the hospital. The experience of the hospital stay was investigated using a visual analogic scale question ranging from 0 (bad) to 100 (good).

The complications and readmissions were recorded through the app up to 6 weeks after surgery. An independent medical doctor classified the complication according to a standardized list of the Knee and Hip Society [22,23]. Then, the complication was confirmed as related to the surgery or classified as a normal undesirable event. Early and late postoperative complications were assessed by the doctor through careful analysis of the electronic medical record (clinical notes and radiographic images) as well as the ones reported in the application. The early ones that were systematically screened for include nausea and vomiting, swelling, wound problem, infection, DVT or PE, bleeding, nerve palsy, periprosthetic fracture, urinary retention. The screening for late complications included stiffness, ongoing uncontrolled pain, sleep disturbance, limping, infection, DVT or PE, implant loosening.

The unplanned consultations with a health care provider (general practitioner, surgeon) during the first week after surgery were recorded through two questions in the app: “How many times did you consult a health care practitioner last week?” and “Was this a planned or an unplanned consultation?”.

Pain level was measured daily through the app using a visual analogic scale (VAS) [7,10,11,12,13]. Type and frequency of medication uptake was measured daily. The number of days of pain above 4/10 and the number of days of medication consumption were recorded as milestones during the recovery. Finally, the day the patient stopped using crutches or went back to driving a car were also recorded. 

Patient-Reported Outcome Measure (KOOS or HOOS) were recorded preoperatively, at 6 weeks and 3 months postoperatively using the app. Satisfaction was assessed using the Knee Society Satisfaction Score and a custom-made questionnaire at 6 weeks and 3 moths postoperatively. 

### 2.2. Statistics

Descriptive statistics were performed for all outcomes. The patient-reported outcomes and the pain medication uptake of the two matched groups were compared with a Mann–Whitney U test. An alpha error threshold of 0.05 was used.

## 3. Results

### 3.1. Participants

The balanced distribution of patients’ demographics and characteristics after matching is detailed in Table 1. The matching resulted in 50 pandemic hip patients matched to 50 pre-pandemic hip patients based on the similarity between their propensity score. Similarly, 41 pandemic knee patients were compared to their most closely matched prepandemic control patient.

No difference was observed between the two groups. The ASA score distribution was the same in both groups.

### 3.2. Length of Stay (LOS)

**Figure 2:**
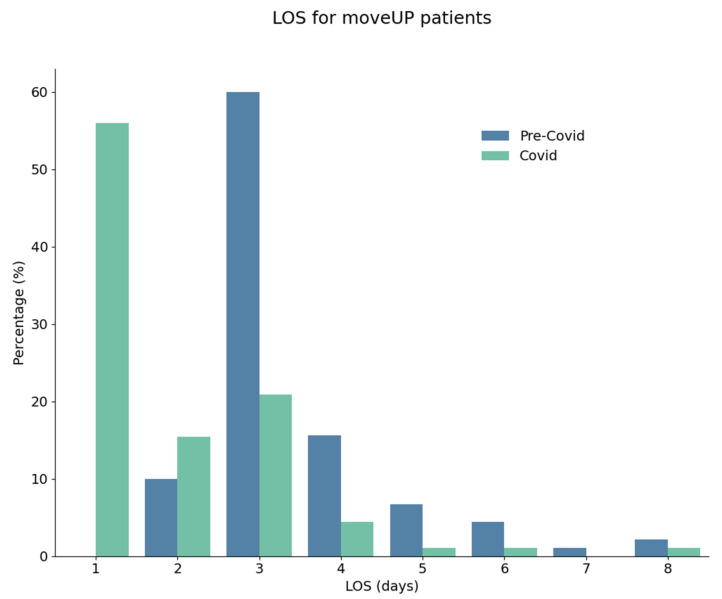
Repartition (%) of length of stay duration (days) for knee and hip surgeries before and during the pandemic.

Before the pandemic, 66% of the patients spent 3 nights in hospital. During the pandemic, 54% of the patients spent only 1 night at hospital (Figure 2). The median value changed from 3 days (interquartile space 3–4) to 1 day (interquartile space 1–3). This difference was statistically significant (<0.001). No differences were observed for the hospital stay experience (87/100) for both groups assessed on a visual analogic scale from 0 (bad) to 100 (good).

### 3.3. Complications, Readmissions and Unplanned Consultations

Overall, 30 events were reported amongst the 292 patients, which gives a patient-reported undesirable event rate of 10%. Of the 30 patient’s reported event, only six were considered as true surgical complications based on the American Hip and Knee classification, and the rate was similar (2%) for the pre-pandemic and pandemic period (pre-pandemic: 4/194 patients, pandemic: 2/97 patients). 

The following complications were recorded during the pre-pandemic phase: knee stiffness (no manipulation needed), implant fracture (requiring exchange of the liner), wound complication (in a diabetic patient, treated conservatively with good results) and skin infection.

The following complications were recorded during the pandemic phase: one deep infection (acute hematogenous infection requiring DAIR procedure with a good outcome), one hip dislocation (requiring reduction in emergency).

During the pandemic, 5.5% of unplanned consultations occurred during the first week after surgery, while there were none during the pre-pandemic period. 

### 3.4. Pain and Medication

**Figure 3:**
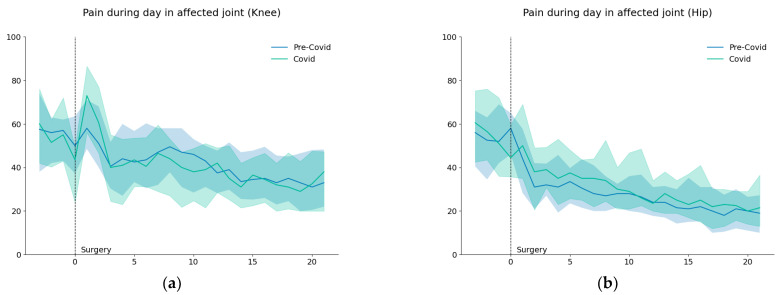
Average and 25–75 interquartile range of pain (VAS) for knee (**a**) and hip patients (**b**) before and twenty days after surgery.

Response rates to the daily questionnaire were 78% for the pandemic group and 85% for the pre-pandemic group. Average pain levels are shown in Figure 3. 

**Figure 4:**
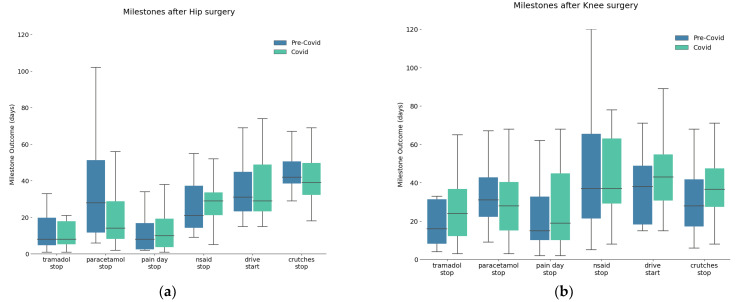
Medication consumption represented by the number of days of painkillers use, and activity recovery represented by the number of days of crutches use and day the patient went back to driving (if relevant) for knee (**a**) and hip (**b**) patients.

**Figure 5:**
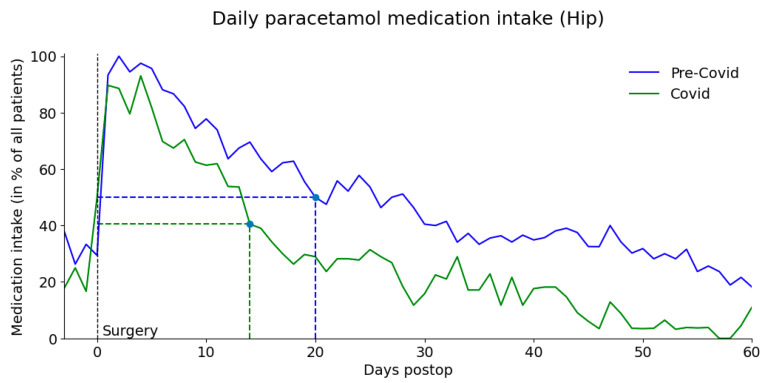
Fraction of hip patients receiving paracetamol. The horizontal line indicates 50% (half of the patients) in each group and the vertical line indicates the postoperative day on which half of the patients stopped consuming paracetamol.

There were no statistical differences for medication consumption except for delay to stop the paracetamol: pandemic group stopped taking paracetamol earlier (Figure 4). The average number of days of paracetamol use decreased from 28 to 14 days during the pandemic period (*p* = 0.004) (Figure 5). Forty-two days after surgery, 52% of knee patients and 32% of hip patients were still taking analgesics in the pandemic group while 69% of knee patients and 50% of hip patients were still taking analgesics in the pre-pandemic group. 

### 3.5. Patient-reported Outcomes

The matched analysis showed no statistical difference for the HOOS, KOOS, satisfaction and several milestones between the pre-pandemic and the pandemic period at any of the time points (Table 2). 

## 4. Discussion

In Europe, the pandemic had a major impact on the epidemiology of hip and knee arthroplasties [5]. To be able to keep operating on patients for THA and TKA, surgeons had to shift from a standard to a short length of stay very quickly for their patients. Shifting quickly to a shorter LOS requires usually time-consuming changes in surgeon’s practice, preparation and teamwork, with progressive implementation of the new pathway within the institution. It was our hypothesis that a quick change from a three- to one-night stay could be implemented safely and efficiently thanks to a dedicated digital follow-up tool, without the need of additional inpatient hospital visits or training, which were not possible during the pandemic. The main goal of this retrospective study was to compare the results in terms of complications measured as the rate of readmission and complications. The second goal was to evaluate the pain management and control. The third goal was to compare the patient-reported outcomes before and after the implementation of the outpatient surgery. The results of this study showed that after hip or knee arthroplasties, decreasing LOS to one day using a mobile health application resulted in a stable rate of complications, readmissions and comparable clinical outcomes. 

This study has several limitations. First, the retrospective design and the limited number of patients limit the impact of the results. Randomized controlled trials are warranted to assess the impact of reduced LOS conclusively. Second, only patients using the digital telerehabilitation were included, as the same level of detailed data is not collected for other patients. This may limit the external validity of our findings. However, the selection criteria for inclusion in the analysis were similar in both groups and patients were matched in both groups, therefore eliminating selection bias between groups. Third, we hypothesized that the digital follow-up tool contributed to reducing the LOS quickly, but its impact cannot be separated from the multidisciplinary effort of the care team in the hospital. The lower workload of the care team of the hospital, enabled by the digital solution, has not been assessed. The exact impact of the digital tool in fast-track programs needs to be studied in a proper prospective study. Despite these limitations, this study was a comparative mono-centric matched-pair study with significant groups of patients allowing comparison. 

The rates of readmission and complications were similar before and after implementing outpatient surgery. The types of complication that occurred were not linked to the changes implied by the fast-track program and could not have been averted by shorter or longer length of stay. This result is in line with literature reporting on fast-track pathways in elective hip and knee arthroplasty. The novelty of this study relies on the use of the digital follow-up tool to shift quickly to outpatient surgery. A lot of criteria are usually needed to achieve outpatient surgery and preparation time is often required for clinical teams [13]. The hospital team followed the criteria given by the authorities to select the patient candidates for surgery (ASA1 and 2), patient not alone at home. The medical dashboard of the digital follow-up tool helped each team member to gain insight into each other’s fields and thus plan the best possible care for their patients. Our hypothesis was verified: the digital follow-up tool helped to quickly shift from standard hospitalization to one-day length of stay. The other benefits related to the use of the digital tool were the reduction of postoperative cost related to in-patient physical therapy sessions and potential reduction of emergency department visits [24,25]. The important difference between the patient complaints (when they actually think that they have a complication) and the rate of what can actually be considered as a complication (confirmed by an independent surgeon) demonstrated that the precise rate of complication may not be adequately estimated from patient-reported data [16]. Patients are likely to over-report problems consecutive to a normal and common increase of stress. The patients indeed tended to report all potential problems, such as minor wound issues, stiffness or unexpected pain [26]. The digital follow-up tool (including the ability to send pictures and videos) allowed us to monitor patient questions about wound care, painkiller medication or swelling, and to reassure them, which limited unnecessary consultations [27]. The tools also allowed us to detect real complications which were confirmed by looking at the medical records. It also helped to deliver an early and efficient treatment, which is also a great advantage of the digital tool.

Concerning the pain management and medical consumption, the results were similar in the two groups. Studies have shown that patients using digital rehabilitation tools have a better early functional outcome and less pain [28,29]. This was not the main goal of this study, but showing that patients were not experiencing more pain when discharged earlier thanks to the digital pain management tool was a confirmation of the efficiency of the digital tool. Pain evolution after TKA is not linear, highlighting the need of good pain control at precise moments [30]. The close monitoring of symptoms through a digital follow-up tool was an efficient way to personalize the treatment, particularly regarding the pain levels. The use of the wearable and the exercises given through the digital follow-up tool also helped to achieve early mobilization, which was managed by the caregiver remotely through the mobile messaging system. Wearables have proven to be effective for patient’s education and patient’s engagement [28,31]. Time and nursed time burden was avoided by, for instance, implementing multiple education and expectation management tasks within the information modules of the application. The results of the latest systematic review [13] were confirmed by this study, showing that patient-reported outcomes were similar for the outpatient group during the pandemic. Despite a shorter LOS, the patients were satisfied. Going back home earlier was comforting during this anxious period in hospital. Previous studies have already shown that patients had similar satisfaction levels when using digital rehabilitation [32,33,34]. The reduced need for transportation, the remote relationship, the personalized treatment and the ease of use are some of the advantages of digital rehabilitation [35]. 

The COVID-19 pandemic, due to the combination of a important backlog of surgeries and strict policies of cost-management, to a regain of interest in outpatient arthroplasty in Europe, in order to optimize the utilization of health care resources [19,36]. Outpatient surgery is still underutilized [37]. Only 0.5% of all total hip and total knee arthroplasties (THA and TKA) in UK in 2018–2019 were performed as outpatient procedures [37]. After the COVID-19 pandemic, the rate of outpatient surgeries is progressively growing, but is still far from the US level. In Europe, due to the reimbursement format, there is no excess of push for true outpatient surgery (discharge the same day). The trend is, however, to decrease the length of stay to one night after surgery and optimize home-care physiotherapy programs. In this context, smart digital-based PT programs are a very important tool to guarantee a continuous monitoring of the patients and improve functional results and safety after hip or knee surgery [38]. Rapid recovery is a multidisciplinary team effort. The staff involved in fast-track surgery often need time to adapt their work philosophy [39]. There is often fear concerning organizational aspects: patients might need more help at home instead of being cared for in hospital, additional work for remote follow-up, or a critical transfer to general practitioners. The support of a digital follow-up tool is a potential solution for the better management of the transition to shorter length of stay. 

Our data suggest that a rapid transition to a shorter LOS, from a three- to one-night stay, using a digital follow-up tool is safe and results in good clinical outcomes. Patients were properly followed up and guided when discharged home. The personalized digital follow-up performed by an experienced team through the tool contributed to these results. Indeed, the smart tool allowed a close monitoring of the patient’s recovery, early identification of possible complication and consecutively immediate intervention. The concept used in this study could be transferred to other centers with limited investments, and may have significant economic implications thanks to reduced hospital costs [40]. This hypothesis should be confirmed in prospective multicentric medico-economic studies. 

## Figures and Tables

**Table 1:** Patients’ demographics.

	Pre-Pandemic	Pandemic
	2018–2020	2020–2021
	**Hip surgeries**	
n	50	50
Age (SD)	62.8 (9.5)	63.5 (11.8)
Gender (%)	24 Men (48%)	25 Men (50%)
	26 Women (52%)	25 Women (50%)
	**Knee surgeries**	
n	41	41
Age (SD)	64.2 (8.5)	65.1 (10.4)
Gender (%)	25 Men (61%)	29 Men (71%)
	16 Women (39%)	12 Women (29%)

n: number of participants; SD: standard deviation.

**Table 2:** Comparison of PROMS and milestones for Hip and knee surgeries.

		Hip		Knee	
		Pre-COVID	COVID	Pre-COVID	COVID
Preoperative	Sample size (n)	50	50	41	41
	Symptoms	47 [34–60]	45 [30–60]	50 [38–64]	46 [32–59]
	Pain	41 [32–50]	43 [30–53]	44 [30–53]	42 [34–57]
	ADL	40 [32–51]	46 [26–59]	43 [29–51]	44 [34–52]
	QOL	25 [19–38]	31 [13–44]	19 [13–31]	25 [13–38]
6 weeks postop	Symptoms	75 [65–85]	75 [59–85]	64 [54–73]	61 [43–68]
	Pain	78 [68–91]	79 [68–89]	67 [54–78]	67 [50–81]
	ADL	75 [64–87]	71 [62–85]	71 [53–82]	72 [51–84]
	QOL	56 [44–69]	56 [50–76]	50 [38–56]	38 [31–50]
3 months postop	Symptoms	80 [70–95]	75 [65–90]	68 [57–79]	64 [50–79]
	Pain	90 [79–98]	80 [71–95]	75 [66–86]	72 [60–86]
	ADL	88 [66–95]	79 [66–93]	75 [64–87]	74 [57–91]
	QOL	69 [55–88]	66 [50–80]	56 [38–63]	50 [38–69]
Milestones	Crutches stop	42 [38–51]	39 [31–50]	28 [17–45]	38 [27–48]
	Drive start	32 [23–45]	32 [22–50]	39 [18–52]	43 [27–56]
	NSAID stop	21 [14–38]	29 [21–33]	38 [29–64]	37 [21–66]
	Paracetamol stop	28 [11–53]	14 [8–31] *	28 [15–43]	31 [14–64]
	Tradonal stop	8 [4–21]	8 [3–19]	16 [8–32]	24 [11–38]
	Pain day stop	8 [2–22]	9[3–18]	15 [10–33]	21 [9–46]
	Pain night stop	5 [2–22]	6 [3–14]	21 [5–34]	23 [11–39]
Satisfaction	KSS score	-	-	29 [22–32]	26 [18–30]

*: significant difference between pre-COVID and COVID group (*p* < 0.05), Data are presented as median with interquartile ranges. ADL: activities of daily living, QoL: quality of life, NSAID: non steroid anti-inflammatory drugs, Tradonal: opioid medication.

**Figure A1:**
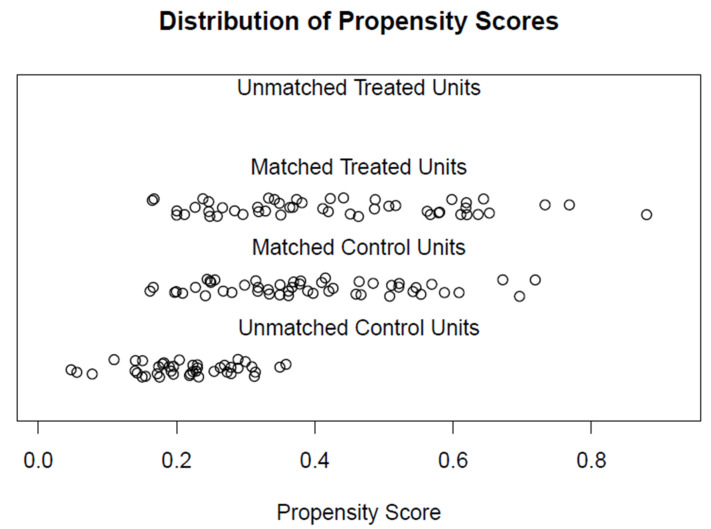
Propensity score matching for Hip patients.

**Figure A2:**
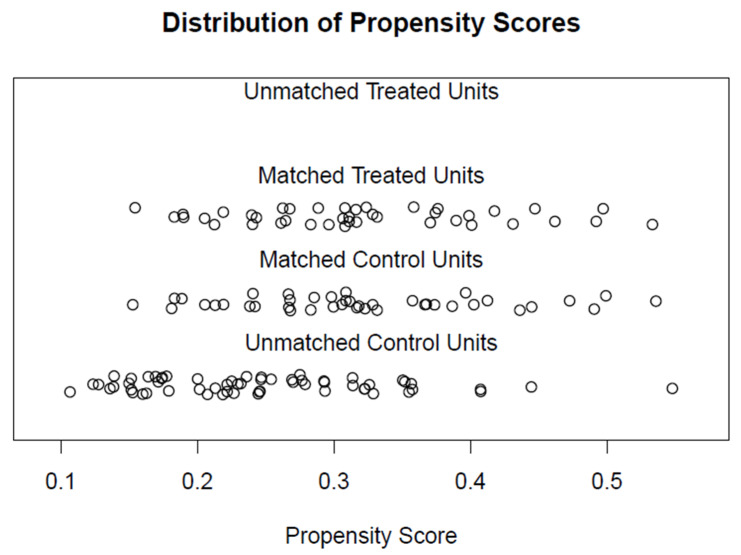
Propensity score matching for Knee patients.

## Data Availability

The data presented in this study are available on request from the corresponding author. The data are not publicly available due to privacy reasons.

## Appendix A. Propensity Scores Matching Results

In order to minimize potential biases and enhance the comparability between the study and control groups, propensity score matching was employed as part of the analysis. Propensity scores were calculated for each patient based on their demographic and clinical characteristics, including age (±5), gender, BMI (Body Mass Index) (±3), type of surgery, pre-operative PROMS (Patient Reported Outcome Measures), and comorbidities measured with the ASA classification American Society of Anesthesiologists physical status classification system) [20,21]. Only ASA 1 and 2 were included. Subsequently, patients from the study group (Post COVID) were matched to corresponding patients from the control group (Pre COVID) based on these propensity scores. This process ensured that individuals with similar profiles were paired together, thereby reducing the impact of confounding variables. The effectiveness of the propensity score matching was confirmed by evaluating the standardized mean differences before and after matching, demonstrating a significant improvement in the balance between the groups. For hip patients (Figure A1), this resulted in a treatment and control group of both 50 subjects. The matching of the knee patients resulted in two groups of 42 subjects (Figure A2). For both hip and knee, all treated units were matched with a control unit. The abundant, unmatched control units were left out of the study.
